# From “immune silence” to “immune dialogue”: modification strategies for bone substitutes based on bone immunomodulatory characteristics

**DOI:** 10.3389/fcell.2025.1685907

**Published:** 2025-10-28

**Authors:** Jiaming Zhao, Quan Sun, Jing Gu, Xiaofan Xu, Minghui Xia, Haibin Xia, Zifan Zhao

**Affiliations:** ^1^ State Key Laboratory of Oral & Maxillofacial Reconstruction and Regeneration, Key Laboratory of Oral Biomedicine Ministry of Education, Hubei Key Laboratory of Stomatology, School & Hospital of Stomatology, Wuhan University, Wuhan, China; ^2^ The Affiliated Tai’ an City Central Hospital of Qingdao University, Tai’ an, China

**Keywords:** osteoimmunomodulation, bone substitute materials, immune microenvironment, biomaterial design, macrophage polarization

## Abstract

Traditional bone substitute materials primarily employ a strategy centered on the direct modulation of osteoblast differentiation. However, this strategy, to some extent, overlooks the pivotal regulatory role of the immune microenvironment in the process of bone regeneration. With the continuous advancement of bone biology research, the significant regulatory role of the immune microenvironment in the osteogenic process has gradually been substantiated. Osteoimmunology studies reveal that immune cells dynamically coordinate the osteoblast-osteoclast balance through shared signaling networks. The “immune-silent” characteristic of traditional bone substitute materials often leads to fibrous encapsulation and failure of osseointegration at the surgical site. Conversely, the research focus of the new generation of bone substitute materials is centered on dynamic immune interaction strategies: by optimizing surface topology to guide macrophages toward a reparative polarization; leveraging the temporal release of bioactive ions to precisely regulate the balance between inflammation and regeneration; and integrating intelligent response systems to dynamically adapt to changes in the pathological microenvironment. Through the synergistic effects of these multifaceted approaches, the ultimate goal is to effectively promote bone tissue regeneration. Against this backdrop, this paper proposes a transition strategy from “immune silence” to “immune dialogue,” which emphasizes the active and effective modulation of immune responses through meticulous material design, thereby reshaping the bone microenvironment to create favorable conditions for bone tissue repair and reconstruction. This innovative concept breaks through the limitations of traditional unidirectional osteogenic modulation, successfully establishing a two-way dialogue bridge between bone substitute materials and the immune system, significantly improving the efficiency of clinical bone defect repair, while also greatly enhancing patient satisfaction. This review systematically outlines the latest advancements in the fields of osteoimmunology and biomaterials, focusing on the key scientific issue of “osteogenic differentiation regulated by the osteoimmune microenvironment,” and provides an in-depth analysis of biomaterial design strategies based on the dynamic balance of the immune microenvironment. The aim is to elucidate the immune-metabolic modulation mechanisms mediated by materials, thereby enhancing the clinical translation efficacy of biomaterials, and provide theoretical support and technical pathways for the precise repair of bone defects.

## 1 Introduction

Bone defects represent a complex clinical pathological condition that poses significant medical challenges in their repair and treatment. Epidemiological data indicate a continuous upward trend in the global incidence of large segmental bone defects, primarily caused by trauma, tumor resection, infection, and congenital developmental anomalies (Zhang and Wang, 2020; Ouyang and Di, 2024). Maxillofacial bone defects not only severely impair patients’ physiological functions, such as mastication and speech, but also lead to abnormal facial contours, significantly affecting patients’ social and psychological wellbeing (Tu et al., 2023). In current clinical practice, while autologous bone grafting is widely recognized as the gold standard for treatment, it is inherently limited by donor site morbidity and restricted graft availability. Conversely, allogeneic bone grafting is associated with risks such as immune rejection and potential pathogen transmission (Schmidt, 2021; Yang et al., 2020). Therefore, the development of bone substitute materials with excellent biocompatibility and osteoinductive activity to facilitate the functional regeneration of bone tissue has emerged as a significant scientific challenge in the field of biomedical engineering. Although substantial progress has been made in optimizing the composition, structural design, and preparation processes of bone substitute materials, their clinical applications continue to face numerous limitations. Firstly, the accelerated aging process of the population has resulted in a sharp increase in cases of osteoporotic fractures and alveolar bone resorption, which raises higher demands on the mechanical properties and biological activity of these materials (Albuquerque-Souza et al., 2022). Secondly, Metabolic diseases, such as diabetes, can induce chronic low-grade inflammatory responses that inhibit the osteogenic differentiation potential of bone marrow mesenchymal stem cells while simultaneously activating osteoclast functions. This ultimately disrupts the homeostasis of the bone repair microenvironment (Zhu et al., 2025). Notably, although traditional bone substitute materials demonstrate good biocompatibility and osteoconductivity, their inability to precisely regulate host immune responses often results in foreign body reactions or fibrous encapsulation, which adversely affects angiogenesis and new bone formation (Barbeck et al., 2015; Chen et al., 2015). These limitations underscore the urgent need to explore the molecular mechanisms of osteoimmunomodulation in greater depth and to develop a new generation of smart bone substitute materials accordingly.

In recent years, significant advancements in bone immunology research have revealed the central regulatory role of the immune system in maintaining bone homeostasis and promoting bone regeneration. This provides a revolutionary theoretical basis for the design of bone substitute materials (Qiu et al., 2020). Studies indicate that the immune system not only participates in the inflammatory response during the initial phase of implantation but, more importantly, regulates the entire bone regeneration process through a sophisticated cell-cytokine network. Immune cells, in conjunction with other cells in the local microenvironment, jointly determine the ultimate outcome of bone repair through complex interactions. Based on these findings, the paradigm for the development of bone substitute materials has shifted from a mere immune evasion strategy to an active strategy of immune microenvironment modulation. By precisely controlling the physicochemical properties of materials, conducting specific functional modifications, and optimizing degradation performance, it is possible to effectively guide the host immune response towards promoting tissue repair (Chen K. et al., 2024; Li C. et al., 2024; Yang Y. et al., 2025). This review will systematically outline the development history of bone defect repair materials, deeply analyze the key role of immune cells in the bone repair process, detail design strategies for bone substitute materials based on immune modulation principles, and anticipate future directions in this field, aiming to provide a solid theoretical foundation and innovative ideas for the development of ideal bone substitute materials.

## 2 The evolution of bone defect repair materials: from “immune silence” to “immune dialogue”

### 2.1 The evolution and limitations of the “immune silence” strategy

Excessive immune responses can lead to chronic inflammation and the formation of a fibrous capsule around the implant. This fibrous encapsulation may hinder direct contact between the bone marrow and the implant, adversely affecting the attachment of bone cells and the generation of new bone, ultimately resulting in the failure of osseointegration (Wegener et al., 2021; Dewey and Harley, 2021). To mitigate adverse immune responses, the development of traditional bone substitute materials has primarily focused on achieving “bioinertness,” aiming for an “immune silence” effect (He et al., 2025). These “immune silent” bone substitute materials are engineered to modulate the bone immune microenvironment, thereby reducing inflammatory responses and excessive activation of immune cells, which constructs a stable microenvironment conducive to bone tissue repair (Liu D. et al., 2024; Wang et al., 2023).

#### 2.1.1 Autologous bone: the clinical gold standard with natural “immune-silent” characteristics

Autologous bone grafting has long been regarded as the “gold standard” for the repair of bone defects since its clinical introduction in the late 19th century, attributed to its osteoconductive, osteoinductive, and osteogenic properties, alongside the absence of immune rejection risks (Schmidt, 2021). The primary advantage of this technique lies in the preservation of natural bone matrix components, including collagen and growth factors, as well as viable cells that actively participate in the bone regeneration process. However, autologous bone grafting is not without complications, such as donor site pain and infection, significant limitations on the quantity of material that can be harvested, and elevated postoperative resorption rates. These challenges considerably limit its clinical applicability, particularly in cases involving large bone defects or the necessity for multiple surgeries (Rönnerfalk et al., 2023; O’Malley et al., 2014). As a result, researchers have begun to explore alternative materials that can fulfill clinical requirements for bone defect repair.

#### 2.1.2 Allogeneic bone: exploration of alternative materials for artificially induced “immune silence”

Allogeneic bone grafts are sourced from the bone tissue of different individuals within the same species. Since Macewen successfully performed the first allogeneic tibial transplantation in a 4-year-old boy with a humeral defect in 1880 (Chen et al., 2010), the field of allogeneic bone transplantation has evolved significantly over more than a century of clinical practice and technological advancements, establishing itself as a vital option to address the limitations of autologous bone (Zhang et al., 2019). Compared to autologous bone transplantation, allogeneic bone grafts provide a wider array of donor sources, encompassing both living and cadaveric donors. This effectively addresses the limitations associated with the scarcity of autologous bone. Furthermore, allogeneic grafts offer considerable advantages in terms of ease of procurement, bone volume, and morphological plasticity. These factors facilitate the rapid reconstruction of blood circulation in bone tissue after implantation, thereby highlighting their significant clinical application prospects (Yang, 2020). Despite the advancements in processing techniques such as deep cryopreservation, freeze-drying, and demineralization aimed at reducing the immunogenicity of allogeneic bone (Liu Z. et al., 2023), this therapy continues to encounter several challenges. These challenges include the presence of residual antigens that may trigger delayed-type rejection reactions, a relatively slow rate of bone healing, the potential risk of disease transmission, and high costs associated with preparation, processing, and storage (Polo-Corrales et al., 2014). Collectively, these factors contribute to a higher failure rate of bone integration in allogeneic bone transplantation, thereby somewhat limiting its further clinical application.

#### 2.1.3 Xenogeneic bone: balancing bioactivity and immunogenicity

Xenogeneic bone materials refer to bone substitute materials derived from non-human species, primarily including bone substitute materials sourced from mammals such as pigs and cattle, as well as corals and algae (Górski et al., 2025). Among these, porcine and bovine bones have become the most extensively studied xenogeneic bone materials due to their accessibility. After undergoing physicochemical treatment to eliminate immunogenicity, xenogeneic bone materials generally exhibit good biocompatibility. Their porous structure provides physical support for host cell migration and vascular ingrowth, thereby promoting osteoconduction (Aleynik et al., 2024). Additionally, by compounding with polymers or adjusting the calcination temperature, the degradation rate of xenogeneic bone materials can be precisely regulated to align with the new bone formation cycle post-implantation, thereby enhancing the success rate of osseointegration (Zhou et al., 2018). Currently, xenogeneic bone materials are widely utilized in clinical applications for the repair of oral and maxillofacial bone defects. For instance, deproteinized bovine bone mineral (DBBM) combined with collagen membranes is employed in guided bone regeneration (GBR) to effectively achieve alveolar ridge augmentation (Sanz-Martin et al., 2018). Furthermore, particulate xenografts, such as deproteinized bovine bone (DBBP), have become popular materials in sinus lift procedures due to their excellent biocompatibility and osteoconductivity (Carmagnola et al., 2024). Their particle size range of 0.25–1 mm not only provides adequate support to the surgical site but also facilitates the formation and integration of new bone (Carmagnola et al., 2024; Wang X. et al., 2016). However, xenogeneic bone materials still exhibit several insurmountable limitations. One significant limitation is the lack of osteoinductivity; due to the absence of active growth factors, such as BMP-2, their osteogenesis process heavily relies on the migratory capacity of host cells. This reliance leads to delayed material absorption, slow new bone formation, and potential failure of osseointegration, particularly in areas with low vascularization (Yao et al., 2022; Chen et al., 2023). Another notable limitation is the insufficient mechanical properties of these materials. Those treated through calcination and oxidant deproteinization display markedly increased brittleness, rendering them inadequate for load-bearing applications within the body (Yang et al., 2018). Consequently, the development of xenogeneic bone materials has yet to fully address the dual challenges of “quality” (mechanical strength) and “quantity” (osteoinductive/osteogenic capacity) that arise during clinical bone defect repair.

In the development process of traditional bone substitute materials, reducing immunogenicity effectively decreases acute rejection reactions; however, it is challenging to avoid chronic inflammation and fibrous capsule formation resulting from long-term foreign body reactions. More importantly, this strategy of “immune silence” compromises the inherent bone regeneration-related bioactivity of these materials, leading to a diminished capacity to regulate the bone immune microenvironment, which ultimately limits the efficiency of bone defect repair. As research in bone immunology has advanced, the academic community has increasingly recognized that ideal bone substitute materials should not only facilitate immune evasion but also possess the ability to actively regulate host immune responses. This understanding has prompted a paradigm shift in research from “immune silence” to “immune modulation”, establishing an immune-osteogenic coupling microenvironment that promotes bone regeneration and enables active control over the bone repair process. This strategy not only overcomes the limitations of traditional materials but also provides a theoretical foundation for the development of a new generation of bone substitute materials that exhibit both immunocompatibility and bone-inducing activity.

### 2.2 “Immune dialogue”: a new development trend in bone substitute materials

Currently, the research strategy for bone substitute materials is shifting from “immune silence” to “immune dialogue.” The term “Immune Dialogue” refers to a bidirectional and dynamic communication process between materials and the host immune system. Specifically, materials can actively regulate the behavior and polarization types of immune cells through their surface properties, degradation products, and ion release, among other physicochemical characteristics. Concurrently, immune cells can influence the degradation rate, surface stability, and release of bioactive substances from materials by secreting cytokines, enzymes (such as MMPs and cathepsins), and ROS. For instance, the acidic environment and proteases secreted by M1 macrophages can facilitate the degradation of certain materials, thereby altering their ion release behavior and bioactivity. Conversely, components such as lactic acid or magnesium ions produced during material degradation can induce the polarization of macrophages towards the M2 phenotype, thus creating a positive feedback loop that collectively enhances the bone repair microenvironment. This bidirectional interaction mechanism distinguishes “immune dialogue” from traditional unidirectional modulatory strategies, more accurately simulating the interplay between the immune system and the microenvironment during natural bone regeneration.

This fundamental transition arises from a deeper understanding within the academic community regarding the physiological processes of bone regeneration. The repair of bone defects is a dynamic process that involves complex interactions among various cells and cytokines, with the immune response playing a central regulatory role (Chen Z. et al., 2017). Studies have shown that indiscriminately suppressing the immune system can hinder bone regeneration. In contrast, early transient pro-inflammatory microenvironments induced by materials such as calcium silicate—characterized by the activation of M1 macrophages—can significantly enhance the osteogenic differentiation and matrix mineralization of bone marrow mesenchymal stem cells (BMSCs). This occurs despite the accompanying increase in reactive oxygen species (ROS), calcium overload, and mitochondrial dysfunction, as it upregulates autophagy-related proteins and pro-inflammatory factors, such as TNF-α (Yu et al., 2024; Luo et al., 2022). Furthermore, TNF-α can upregulate the expression of CD73 in the exosomes of mesenchymal stem cells, enhancing their immunosuppressive function and subsequently promoting the polarization of M2 macrophages. This process coordinates the later stages of the bone healing process (Lu T. et al., 2022). These findings underscore the core concept of immune dialogue: implanted materials must dynamically interact with the host immune system and bone cells to precisely regulate the balance of the inflammatory factor network. This involves suppressing excessive inflammation to prevent fibrous encapsulation and failure of bone integration while providing moderate immune stimulation to induce bone regeneration.

From the perspective of the development history of materials science, the evolution of this concept has progressed through three distinct stages. The first generation of materials, such as hydroxyapatite (HA) and beta-tricalcium phosphate (β-TCP), primarily focused on their fundamental role as bone defect fillers, emphasizing the materials” biocompatibility and mechanical properties (Zhao et al., 2021; Ye et al., 2022). The objective was to restore the defect morphology and provide mechanical support. However, this approach overlooked the dynamic changes occurring in the bone regeneration microenvironment, resulting in limited repair effects that often failed to meet clinical needs (Tran et al., 2025). In contrast, the design philosophy of second-generation materials, including bioactive glass (BG) and calcium phosphate cements (CPCs), shifted towards “biological responsiveness.” This new approach aims to promote bone tissue repair and regeneration more effectively by enhancing the bioactivity of the materials (Dai Q. et al., 2024). The research and development strategy for these materials primarily targets osteoblasts, with the goal of achieving bone regeneration by directly stimulating the differentiation of osteogenic stem cells (Dai Q. et al., 2024; Zha et al., 2015). Nevertheless, osteogenic differentiation is not a process involving a single cell type; it necessitates the collaborative construction of a suitable bone microenvironment by multiple systems (Zhang S. et al., 2025; Hao et al., 2023). In fact, the key to regulating osteogenic differentiation lies in the new microenvironment formed by the interaction between materials and multi-system cells, rather than solely in the role of the materials themselves (Hao et al., 2023; Birmingham et al., 2012). If the importance of other system cells and the microenvironment is overlooked, the developed materials may improperly regulate the microenvironment, potentially hindering or even preventing successful bone regeneration (Campana et al., 2014). Due to the limitations inherent in the development concepts of the first two generations of bone substitute materials, the design of third-generation materials has transitioned towards a strategy of “biological guidance” (Bongio et al., 2010). This approach aims to precisely regulate the release of chemical signals and actively direct cellular behaviors through various means, including surface topology, ion release profiles, and biomolecular modifications, thereby shaping the regenerative microenvironment (Hu D. et al., 2024; Lyu et al., 2025; Zhang S. et al., 2023). This multi-target, multi-scale regulatory strategy enables these materials to effectively circumvent potential “osteoimmunomodulation defects” and establish an “immune dialogue” with host tissues, offering new insights to overcome the current efficacy bottleneck in bone regeneration materials.

## 3 The theoretical basis of osteoimmunology and the role of immune activation in bone repair

### 3.1 Theoretical framework of osteoimmunology

**FIGURE 1:**
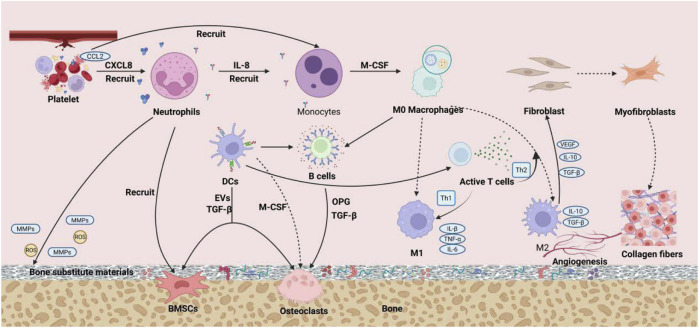
The precise and orderly interactive dialogue between immune cells and stromal cells jointly determines the process of bone defect repair.

Bone immunology, as a cutting-edge interdisciplinary field that integrates immunology and bone biology, reveals the complex bidirectional regulatory mechanisms between the immune system and the skeletal system (Takayanagi, 2015). Specifically, a moderately activated immune response is essential for initiating and sustaining the process of bone tissue repair. The bone immune microenvironment constitutes a highly complex and dynamic system, where precise homeostasis is crucial for maintaining the normal physiological functions of bone tissue. The core mechanisms for maintaining this balance lie in the shared molecules (such as key transcription factors, signaling molecules, and membrane receptors) between the immune system and the skeletal system, which functionally depend on one another (Takayanagi, 2015; Takayanagi, 2021). During the process of bone defect repair, the immune system plays a critical role in the fine modulation of the regenerative microenvironment through multidimensional interactions, where the intricate and orderly dialogues between immune cells and matrix cells collectively determine the repair process of bone defects (Figure 1). This chapter will outline the roles and mechanisms of various immune cells in bone defect repair, aiming to depict a multi-layered regulatory network of immune-bone metabolism coupling.

### 3.2 The regulatory role of immune cells in bone regeneration

Research has demonstrated that immune cells play a pivotal role in regulating the synthesis and release of various bioactive substances, including growth factors, chemokines, and inflammatory mediators (Speeckaert et al., 2023). This coordination is essential for key events in the bone repair process, such as regulating osteoblast differentiation, maintaining a balance in osteoclast activity, suppressing fibrosis, and facilitating the construction of a neovascular network (Zha et al., 2018).

#### 3.2.1 Neutrophils

As the most abundant leukocytes in mammalian blood, neutrophils play a crucial role in immune modulation during bone repair. Their functions extend well beyond the initial inflammatory response, significantly influencing various aspects of tissue regeneration and repair (Zhan et al., 2024). In the early phase of bone injury, neutrophils, as the first infiltrating immune cells, effectively clear damage-associated molecular patterns (DAMPs), including necrotic cells and bone debris, by releasing extracellular traps (NETs). This action alleviates excessive inflammatory responses and lays the groundwork for subsequent repair (Carmona-Rivera et al., 2024). Concurrently, they secrete chemokines such as IL-8 and NAP-2 to recruit monocytes and macrophages, actively shaping the inflammatory microenvironment (Liu Z. et al., 2022; Liu et al., 2025). During the bone regeneration phase, neutrophils directly recruit bone marrow-derived mesenchymal stem cells (BMSCs) to the injury site by secreting stromal cell-derived factor-1 (SDF-1). Additionally, they produce ROS and matrix metalloproteinases (MMPs) to facilitate tissue remodeling and angiogenesis, thereby providing essential support for bone regeneration (Cai et al., 2021; Poh et al., 2022). The immunomodulatory functions of neutrophils are mediated through various interactions. Neutrophils secrete factors, such as IL-6 and neutrophil gelatinase-associated lipocalin (NGAL), which drive macrophages to polarize towards the pro-repair M2 phenotype, thereby enhancing osteogenic differentiation and bone matrix deposition (Wang et al., 2019; Xu et al., 2015). Additionally, neutrophil extracellular traps (NETs) can optimize the phagocytic function of macrophages, further coordinating the repair microenvironment (Fang et al., 2022). In terms of immunometabolism, lactate produced by the glycolytic pathway of neutrophils activates the HIF-1α and mTORC1 pathways, inhibiting the expression of pro-inflammatory factors and significantly promoting M2 polarization of macrophages, which enhances osseointegration and promotes angiogenesis (Luo et al., 2025; Caslin et al., 2021). Notably, neutrophil function may be impaired under pathological conditions, such as chronic inflammation. Targeting the inflammatory site with the immunomodulator FTY720 through surface modification or local delivery strategies can restore neutrophils” bactericidal and pro-repair activities (Michael et al., 2025; Byun et al., 2025). In summary, neutrophils construct an immune microenvironment conducive to bone regeneration through multiple mechanisms, including the clearance of damaged substances, directional cell recruitment, modulation of macrophage polarization, mediation of metabolic reprogramming, and promotion of vascular-tissue remodeling. Their precise modulation provides critical targets for optimizing bone repair strategies.

#### 3.2.2 Macrophage

Macrophages, as a crucial component of innate immunity, are primarily categorized into two polarization types: pro-inflammatory (M1) and anti-inflammatory (M2). These two types function independently yet are interrelated, playing distinct roles at various stages of bone regeneration.

M1 macrophages are predominant during the early phase of bone injury repair, where they clear foreign bodies and bone fragments while secreting pro-inflammatory factors such as TNF-α, IL-1, and IL-6 to regulate bone metabolism (Li Y. et al., 2025). In the bone microenvironment, TNF-α inhibits osteogenesis by suppressing the activity of ALP in osteoblasts and the expression of Runx2 protein (Wang et al., 2018). Concurrently, TNF-α and IL-1 work synergistically to downregulate OPG and promote the expression of receptor activator of RANKL, thereby stimulating osteoclast formation and bone resorption (Zha et al., 2018). Additionally, IL-6 significantly reduces ALP activity, inhibits osteogenic gene expression and mineralization efficiency, and negatively regulates osteogenic differentiation by activating the SHP2/MEK2/ERK and SHP2/PI3K/Akt2 pathways (Kaneshiro et al., 2014). Although sustained M1 inflammation inhibits bone formation, studies have demonstrated its essential role in initiating bone regeneration. The VEGF secreted by M1 cells promotes angiogenesis, which is critical for restoring blood supply, facilitating cell homing, and releasing factors (Schlundt et al., 2018). Furthermore, M1 cells can enhance the osteogenesis of BMSCs through direct contact, the secretion of factors, and exosomes, such as those rich in miR-21a-5p, which can induce osteogenic differentiation of BMSCs (Vallés et al., 2020; Xia et al., 2020; Liu K. et al., 2022). Therefore, M1 macrophages play a dual role in bone remodeling.

M2 macrophages play a dominant role in the middle and late stages of bone healing. They secrete IL-10 and TGF-β, which suppress inflammation and promote tissue repair (Lu Y. et al., 2022; Hosseini et al., 2024). Additionally, they are involved in vascular remodeling through the action of VEGF and induce osteogenic differentiation and bone formation in BMSCs via BMP-2 (Sun X. et al., 2021). Research indicates that inducing macrophage polarization towards M2 after 72 h of co-culture with M1 macrophages and osteogenic precursor cells (MC3T3) significantly enhances osteogenesis, highlighting the importance of the sequential polarization of macrophages from M1 to M2 for effective bone regeneration (Loi et al., 2016).

In summary, M1 and M2 macrophages collaboratively regulate bone repair: M1 initiates early inflammation and repair, while M2 predominates in the later stages of anti-inflammation and tissue remodeling. However, persistent activation of M1 can lead to chronic inflammation and fibrous encapsulation, which hinders regeneration and may induce rheumatoid arthritis-like lesions (Chang et al., 2023). Conversely, prolonged activation of M2 may increase the secretion of pro-fibrotic factors, resulting in excessive scar formation and delayed healing (Meng et al., 2025). Therefore, precisely regulating the temporal polarization of macrophages to achieve early transient M1 activation followed by a timely transition to M2 is crucial for optimizing bone regeneration outcomes and represents an important direction for the future development of bone substitute materials.

#### 3.2.3 T lymphocyte

T cells are essential lymphocytes in the adaptive immune system. Numerous studies have confirmed that various cytokines and growth factors secreted by T cells play a significant role in the bone repair process. Moreover, the regulatory effects of T cells on the balance between osteogenesis and osteoclastogenesis display notable differences due to the distinct functions of their subsets.

Th1 and Th2 cells are two major subsets of CD4^+^ T cells. Th1 cells primarily exert their functions through the secretion of IFN-γ and TNF-α, while Th2 cells play a role in immune modulation by secreting IL-4 and IL-10. The roles of Th1 and Th2 cells in bone repair remain controversial. Early studies suggested that Th1 cells promote osteoclast differentiation and bone resorption by expressing RANKL (Kotake et al., 2005). In contrast, Th2 cells enhance the osteogenic differentiation of BMSCs through IL-4 secretion and inhibit Th1-mediated inflammation, thereby optimizing the microenvironment for bone regeneration (Li R. et al., 2024). However, subsequent research has revealed a more complex regulatory mechanism. Researchers Sato et al. (2006) discovered that both IFN-γ and IL-4, secreted by Th1 and Th2 cells, can inhibit osteoclast differentiation and reduce pathological bone resorption under inflammatory conditions by promoting the degradation of tumor necrosis factor receptor-associated factor 6 (TRAF-6), a key adaptor protein in the RANKL/RANK signaling pathway, thereby blocking the activation of this pathway. This finding indicates that the function of Th1 cells is dualistic; their ultimate manifestation as pro-bone resorption or anti-bone resorption may depend on the state of the local microenvironment.

In terms of regulatory T cells (Treg), this immunosuppressive subpopulation directly inhibits the differentiation of osteoclast precursors by secreting cytokines such as IL-4 and TGF-β. Simultaneously, it activates the Wnt/β-catenin signaling pathway through the upregulation of WNT10b expression, thereby promoting the expression of osteogenesis-related genes and achieving bidirectional modulation (Tyagi et al., 2018). Studies have demonstrated that Treg can not only directly inhibit the differentiation of peripheral blood mononuclear cells into osteoclasts (Luo et al., 2011), but also indirectly optimize the bone repair microenvironment by regulating macrophage polarization (Aurora et al., 2014).

The traditional view posits that Th17 cells, a significant subset of CD4^+^ T cells, not only directly promote osteoclast formation through the expression of RANKL in synergy with Th1 cells (Bhattacharya et al., 2023), but also play a crucial role via the secretion of IL-17. On one hand, IL-17 significantly upregulates RANKL expression on the surfaces of osteoclast precursor cells and osteoblasts, thereby promoting osteoclastogenesis while inhibiting osteoblastic differentiation (Li et al., 2019). On the other hand, IL-17 recruits and activates other immune cells, elevating levels of IL-1 and TNF-α in bone tissue, which creates an osteoclast-activating microenvironment that directly impedes new bone formation (Adamopoulos and Bowman, 2008; Li et al., 2022). This dual effect of “promoting osteoclasts while inhibiting osteoblasts” positions Th17 cells as key regulators in inflammatory bone diseases. However, some studies have indicated that IL-17 can synergistically stimulate the proliferation, migration, and osteogenic differentiation of mesenchymal stem cells in conjunction with BMP-2, thereby facilitating new bone formation (Croes et al., 2018; Croes et al., 2016). Consequently, the role of Th17 cells in bone regeneration remains contentious and warrants further investigation.

#### 3.2.4 B lymphocyte

B cells originate from hematopoietic stem cells in the bone marrow and migrate to the spleen and lymph nodes upon maturation. As a crucial component of adaptive immunity, B cells primarily combat pathogens by synthesizing and secreting antibodies. In addition to their immune functions, B cells play a significant role in bone repair. Research indicates that B cells inhibit osteoclast differentiation by secreting OPG, which antagonizes the RANKL signaling pathway (Titanji, 2017). Moreover, they induce osteoclast apoptosis through the release of TGF-β (Weitzmann et al., 2000), thereby exerting a protective role in maintaining physiological bone homeostasis. Furthermore, B cells support the differentiation of BMSCs by interacting with them within the bone microenvironment (Zegallai et al., 2022). However, In pathological conditions such as rheumatoid arthritis, activated B cells can secrete pro-inflammatory factors, including TNF-α and CCL3, as well as inhibitory molecules like sclerostin (SOST) and Wnt signaling pathway inhibitor 1 (DKK1). These factors inhibit the osteogenic differentiation of BMSCs by directly suppressing the activity of the key osteogenic transcription factor RUNX2 or by interfering with the Wnt signaling pathway (Wagner et al., 2017; Katoh and Katoh, 2017; Colucci et al., 2011; Gunn et al., 2006; Zhang et al., 2015). Furthermore, studies have demonstrated that in a periodontitis model, the Gram-negative anaerobic bacterium Tannerella forsythia can induce B cells to significantly upregulate RANKL expression, thereby promoting osteoclastic bone resorption (Settem et al., 2021). In summary, the dual regulatory effect of B cells on osteogenesis is highly dependent on the state of the local microenvironment; an imbalance in this homeostasis may lead to inflammatory bone destruction or metabolic bone disease.

#### 3.2.5 Dendritic cells

Dendritic cells (DCs) are currently recognized as the most potent antigen-presenting cells and play a crucial immunomodulatory role in bone repair. DCs significantly influence the outcomes of bone repair by inducing an immune-tolerant microenvironment, coordinating cellular interactions, and transmitting tissue regeneration signals. Research indicates that DCs can be activated by the local microenvironment, such as magnesium ions via the TRPM7 channel, which triggers the MAPK/HIF-1α/TGF-β signaling axis. The upregulation of HIF-1α promotes TGF-β secretion, subsequently suppressing effector T cell function and expanding regulatory T cells, thereby effectively alleviating bone inflammation and creating a pro-repair microenvironment (Dai Y. et al., 2024). Additionally, the extracellular vesicles (EVs) secreted by DCs carry signaling molecules, including osteopontin (OPN) and matrix metalloproteinase- 9 (MMP-9), which actively recruit BMSCs to the injury site and directly promote bone regeneration (Silva et al., 2017). Recent studies have further revealed that the protein adsorption behavior on the surface of implant materials, particularly the formation of a biomolecular layer dominated by fibronectin (Fn) and HMGB1, can regulate the immune recognition process of DCs, thereby influencing the osteoinductive process mediated by these cells (Zhao et al., 2024; Zhao et al., 2023).

The dynamic interactions between DCs and bone microenvironment cells finely regulate the balance of bone repair. Under the stimulation of RANKL and M-CSF, DCs can differentiate into osteoclasts, thereby participating in bone remodeling (Puchner et al., 2024). Conversely, in conditions characterized by infection or chronic inflammation, TGF-β secreted by DCs can inhibit osteoblast differentiation (Wu et al., 2025; Yang et al., 2024). However, during the immune-suppressed state prevalent in the bone repair phase, TGF-β can mitigate pathological bone destruction, such as by inhibiting the pro-inflammatory effects of the Th17/IL-17 axis in rheumatoid arthritis, thus promoting bone repair (Dai Y. et al., 2024). Notably, DCs exhibit significant potential in tissue engineering: BMSCs differentiated into cartilage do not induce DC maturation or provoke immunogenic responses when co-cultured with DCs, indicating a reduced risk of immune rejection in allogeneic transplantation (Kiernan et al., 2018). Furthermore, the mechanism by which DCs recruit BMSCs through EVs offers valuable insights for the development of novel tissue regeneration strategies (Silva et al., 2017).

#### 3.2.6 Other immune cells

Other innate immune cells also play significant roles in the process of bone repair. For instance, mast cells exhibit spatiotemporal distribution characteristics that are closely related to their functions (Banovac et al., 1995). Following trauma or fracture, mast cells rapidly respond by releasing mediators such as cytokines and chemokines, which initiate and modulate the early inflammatory response, recruit endothelial cells to promote angiogenesis, and provide support for repair (Fischer et al., 2020; Ramirez-GarciaLuna et al., 2017). However, the activity of mast cells must be precisely controlled; excessive activation, as seen in cases of multiple trauma or estrogen deficiency, can exacerbate inflammation, promote the formation of fibrotic scars (Fischer et al., 2020; Ragipoglu et al., 2022), and inhibit osteoblast differentiation while enhancing osteoclast activity through the release of specific mediators such as Midkine and CXCL10 (Fischer et al., 2020). This impairment can negatively affect the quality of bone formation and remodeling. The dual role of mast cells in promoting repair, such as angiogenesis, and inducing disorders, including inflammation, fibrosis, and disruption of bone metabolism, positions them as a highly potential therapeutic target (Zhang et al., 2017). Targeted modulation of mast cell activity is anticipated to optimize bone repair outcomes, particularly in cases of osteoporosis-related bone repair disorders.

Natural killer (NK) cells are pivotal immune regulators in the initial phases of bone repair, primarily by modulating the behavior of mesenchymal stem cells (MSCs) and inflammatory responses. Research indicates that NK cells significantly enhance the invasive capacity of MSCs, promoting their recruitment to injury sites. This process can be optimized through biomaterial design; for instance, functionalized chitosan membranes adsorbed with fibrinogen (Fg) can enhance NK cell adhesion, thereby improving MSC recruitment efficiency (Almeida et al., 2012). Regarding inflammation modulation, early activation of NK cells serves as a “double-edged sword”: moderate activation can coordinate the inflammatory microenvironment to support regeneration, while overactivated NK cells may directly damage normal tissues by releasing substantial amounts of cytotoxic granules (such as perforin and granzymes), which can hinder the repair process (Almeida et al., 2012; Thacker et al., 2023). Notably, in an immunosuppressive environment, the cytotoxic ability of NK cells may be constrained; nevertheless, their role in bone repair remains significant. For example, studies have shown that in immunodeficient mice lacking T cells, MSCs can successfully induce ectopic bone formation, suggesting that the role of NK cells in immune modulation may compete with that of other immune cells, such as T cells (Dighe et al., 2013). Furthermore, when the activities of both IFN-γ and T cells are suppressed, the regulatory role of NK cells becomes more pronounced, enhancing the osteogenic differentiation capability of MSCs. In summary, the design of functional biomaterials targeting the interaction between NK cells, MSCs, and inflammatory regulatory pathways offers new strategies for bone regeneration therapy.

## 4 Design strategy for bone substitute materials based on immune modulation

### 4.1 Design and control of material physicochemical properties

In recent years, a substantial body of research has focused on the immunomodulatory properties of bone substitute materials. The central concept of designing “immunomodulatory” materials involves endowing bone substitute materials with osteoimmunomodulatory characteristics through various modification strategies. This enables effective intervention and precise manipulation of the osteoimmune microenvironment within the host, thereby fostering a bone microenvironment conducive to tissue regeneration (Zhou et al., 2024). The diverse physicochemical properties of the materials, including surface characteristics, mechanical properties, and morphology, significantly influence the local immune responses they elicit.

#### 4.1.1 Surface properties

**FIGURE 2:**
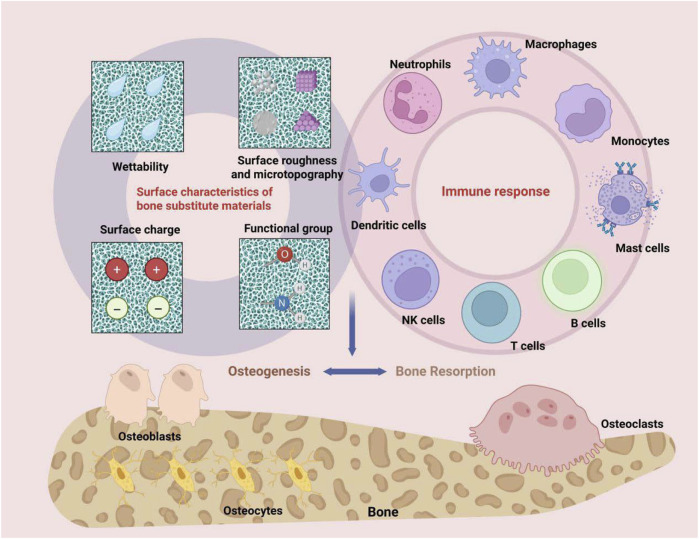
The surface properties of bone substitute materials, such as hydrophilicity/hydrophobicity, roughness, micro-morphology, as well as surface charge and functional groups, can modulate the bone immune environment.

After a bone substitute material is implanted into the tissue, its surface comes into direct contact with the surrounding immune environment, leading to various reactions. The surface properties of the material, including hydrophilicity/hydrophobicity, roughness, microtopography, surface charge, and functional groups, significantly influence the host immune response following implantation by modulating protein adsorption, cell adhesion, and immune cell activation (Figure 2) (Zhou et al., 2024; Ishihara et al., 2020).

Generally, hydrophilic surfaces form dense hydration layers that significantly reduce non-specific protein adsorption (Zheng et al., 2010; Kim et al., 2005). Conversely, hydrophobic surfaces promote protein enrichment through hydrophobic interactions (Guo et al., 2016). The synergistic effect of surface charge and hydrophilicity/hydrophobicity further determines the composition of proteins adsorbed on the material surface. For instance, positively charged hydrophilic surfaces tend to adsorb Fn, while hydrophobic surfaces are inclined to enrich albumin (Alb) (Arima and Iwata, 2007; Wei et al., 2009). In terms of cell adhesion, moderately hydrophilic surfaces have been shown to be the most conducive to cell spreading and adhesion. Taking the self-assembled monolayer (SAM) of alkanethiol as an example, surfaces with hydroxyl (OH) and methyl (CH_3_) groups exhibit optimal cell adhesion performance within a water contact angle range of 40°–50° (Arima and Iwata, 2007; Hasan et al., 2018a). This enhancement is partly due to the ability of hydrophilic surfaces to specifically adsorb cell adhesion proteins (such as Fn) and maintain their suitable conformation (Wei et al., 2009; Hasan et al., 2018a). In contrast, highly hydrophobic surfaces (such as nano-PTFE) severely inhibit the adsorption of cell adhesion proteins, resulting in limited cell spreading and spherical deformation of cell morphology (Zheng et al., 2010; Ainslie et al., 2007). In the realm of immune modulation, hydrophilic surfaces demonstrate superior efficacy in modulating the recruitment of Tregs, diminishing the adhesion of immune cells, and facilitating the polarization of macrophages towards the M2 phenotype. This process significantly reduces the release of pro-inflammatory factors, such as TNF-α and IL-1β, thereby alleviating local inflammatory responses and fostering a microenvironment conducive to bone regeneration (Rmaidi et al., 2021; Zhang et al., 2020). The low immunogenicity associated with hydrophilic surfaces is partially attributed to their capacity to inhibit non-specific protein adsorption. In contrast, hydrophobic surfaces markedly enhance immune cell activation and inflammatory responses, primarily due to their promotion of the adhesion of a substantial number of proteins, including immunoglobulins and platelets (Hu et al., 2016). Notably, the application of surface engineering strategies to impart superhydrophilicity to hydrophobic materials, such as the introduction of zwitterionic groups, can effectively mitigate their immunogenicity and significantly enhance biocompatibility (Chen S-H. et al., 2017).

The surface roughness and microtopography of materials are critical factors in modulating the bone immune microenvironment. Typically, rough surfaces enhance protein adsorption by increasing the contact area between the material and the microenvironment, thereby providing a foundation for cell adhesion (Asadullah et al., 2021). Research has demonstrated that the incorporation of hydroxyapatite (HA) particles onto the surface of polylactic acid (PLA) composites significantly improves cell spreading, the formation of actin stress fibers, and the expression of focal adhesion proteins, all of which are essential markers of cell adhesion (Persson et al., 2014). Similarly, polyetherketoneketone (PEKK) materials embedded with silicon nitride (SN) and tantalum (Ta) microparticles also markedly enhance the adhesion and proliferation of BMSCs by increasing surface roughness and hydrophilicity (Hu et al., 2021). Furthermore, the roughness of the material surface can influence macrophage polarization. For instance, researchers such as Hamlet et al. (2019). Found that titanium implants modified to have rough surfaces significantly accelerate the healing process and greatly enhance the success rate of implantation. This is primarily due to the fact that roughened titanium surfaces more effectively promote the polarization of macrophages towards the M2 phenotype, thereby increasing the expression of anti-inflammatory factors IL-4 and IL-10, and creating a microenvironment conducive to bone regeneration. This effect may arise from the ability of rough surfaces to facilitate extracellular matrix (ECM) deposition and remodeling, or to activate specific signaling pathways (such as the TGF-β and IL-10 pathways) (Persson et al., 2014; Miguel et al., 2010). The microscopic morphology of material surfaces plays a crucial role in influencing the bone immune microenvironment, primarily due to factors such as crystal structure, particle distribution, size, and surface texture. On one hand, the crystal structure significantly affects protein adsorption behavior. For instance, the nanoscale roughness of a silica surface can alter the adsorption orientation of fibrinogen, enhancing its prominence in solution and increasing its bioavailability (Hyltegren et al., 2020). On the other hand, particle distribution and size profoundly influence cell behavior by determining surface roughness and porosity. For example, the microporous structure of β-tricalcium phosphate (β-TCP) particles significantly promotes cell adhesion and bone regeneration (Piccinini et al., 2016). Additionally, surface texture primarily regulates immune responses indirectly. For instance, nanostructured surfaces can effectively reduce bacterial adhesion, thereby lowering the risk of infection (Bhattacharjee et al., 2023). Furthermore, PVA/EPB composites with specific textures exhibit anti-inflammatory and antibacterial properties through their nanofiber structures, further demonstrating the potential of surface textures in immune modulation (Allafchian et al., 2025).

The design of surface charges and functional groups on materials represents a crucial regulatory strategy. Generally, cationic particles on charged surfaces are more likely to induce inflammatory responses compared to anionic particles. This phenomenon may be attributed to the propensity of cationic particles, owing to their positive charge, to engage in electrostatic interactions with negatively charged biomolecules (Valverde-Mendez et al., 2025). For instance, a study revealed that positively charged nanoparticles exhibit a significantly higher binding capacity with NETs than their negatively charged or neutral counterparts, which may exacerbate inflammatory responses and lead to tissue damage (Raghavan et al., 2025). Furthermore, an *in vitro* investigation into the influence of surface charge on cytokine secretion, utilizing a co-culture of monocytes and macrophages, demonstrated (Brodbeck et al., 2002) that the expression of the anti-inflammatory factor IL-10 secreted by these cells was significantly upregulated on anionic surfaces, whereas it was downregulated on cationic surfaces. The technique of modifying functional groups, such as amino and hydroxyl groups, on scaffold surfaces through molecular grafting is an emerging surface modification approach. Functional groups present on the material surface can significantly influence protein adsorption behavior, thereby modulating surrounding cell responses and promoting new bone formation (Hasan et al., 2018b). For example, *in vitro* experiments have confirmed that carboxyl-modified surfaces can encourage the polarization of macrophages towards the M2 phenotype, stimulate anti-inflammatory responses, and significantly enhance the osteogenic differentiation of BMSCs (Buck et al., 2022).

#### 4.1.2 Mechanical properties

The stiffness and elastic modulus of bone substitute materials serve as critical mechanical signals that profoundly reshape the immune microenvironment through mechanotransduction pathways, directly regulating the bone regeneration process.

Research indicates that material stiffness exhibits a biphasic threshold effect on macrophage polarization (Xu et al., 2022; Dutta et al., 2020): when the elastic modulus is within the low modulus range (e.g., 76 kPa collagen hydrogel), an increase in stiffness significantly promotes the dominant expression of the anti-inflammatory M2 phenotype, thereby enhancing the angiogenic capacity of endothelial cells and the osteogenic differentiation ability of BMSCs (Zhang et al., 2024; Sridharan et al., 2021). In contrast, high modulus matrices (e.g., 295 kPa materials) strongly induce pro-inflammatory M1 polarization (Zhang et al., 2024), which not only exacerbates the secretion of pro-inflammatory factors like TNF-α and IL-6 but also promotes macrophage fusion to form foreign body giant cells (FBGCs), triggering a chronic inflammatory cascade that adversely affects the material”s performance and long-term integration (Meli et al., 2021). However, due to inherent limitations in the material”s properties, the range of stiffness effects, and the heterogeneity of experimental models, the mechanism by which stiffness regulates macrophage polarization remains significantly complex, necessitating quantitative analysis through cross-scale *in vivo* and *in vitro* studies (Tang et al., 2021).

#### 4.1.3 Material morphology

**FIGURE 3:**
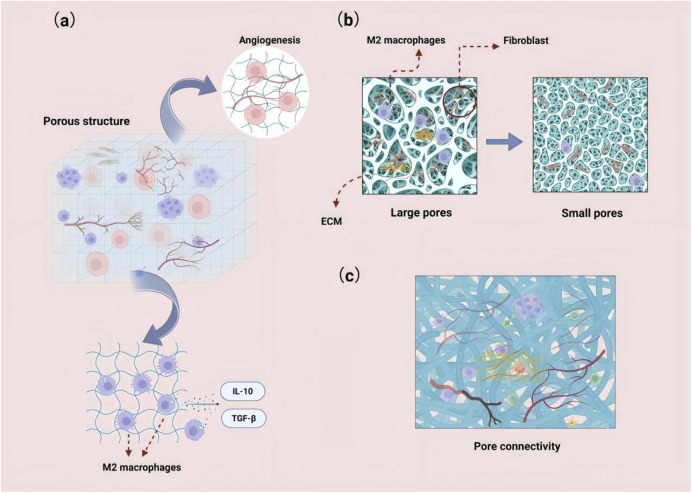
**(a)** Porous structures can provide three-dimensional cell attachment sites and migration pathways, significantly promoting cell infiltration. The moderate hypoxic microenvironment induced by these structures can synergistically enhance the polarization of macrophages to the M2 phenotype and promote angiogenesis. **(b)** Larger pore sizes facilitate the deep infiltration and homogeneous distribution of fibroblasts, and they enhance angiogenesis and extracellular matrix deposition. **(c)** Highly interconnected scaffolds can establish a continuous nutrient/cell transport network.

The morphological modification strategies of bone substitute materials, particularly those focusing on porous structure, pore size, and connectivity, are crucial for enhancing bone regeneration and biocompatibility (Figure 3). The porous structure provides three-dimensional cell attachment sites and migration channels, significantly promoting cell infiltration and osseointegration (Liu et al., 2016). For instance, magnesium phosphate cement (MPC) with high porosity, constructed using a citric acid/calcium carbonate (CaCO_3_/CA) foaming agent, effectively enhances the osteogenic differentiation activity of human periodontal ligament stem cells (hPDLSCs) (Chen et al., 2025). This enhancement is primarily due to the optimized pores that facilitate nutrient diffusion and provide a larger space for cell infiltration (Chou et al., 2013). Furthermore, the porous structure not only regulates cell infiltration behavior but also plays a pivotal role in bone regeneration by mediating immune responses. Research indicates that the porous structure serves as a physical foundation for macrophage adhesion and migration, significantly modulating the intensity of inflammation, osteoclast activity, and the secretion of osteogenic factors, such as BMP-2, by activating key components of the autophagy pathway (Liu J. et al., 2023). Additionally, the moderately hypoxic microenvironment induced by porous structures and large-pore scaffolds can synergistically promote M2 polarization of macrophages and angiogenesis, thereby remodeling the pro-regenerative immune microenvironment (Wan et al., 2025).

Pore size directly influences cell behavior: larger pores (e.g., 100–150 μm) significantly enhance deep infiltration and promote a homogeneous distribution of fibroblasts, whereas smaller pores (e.g., <50 μm) hinder cell surface adhesion (Vieira et al., 2024). By incorporating glucose crystals to increase pore size, PLA electrospun membranes achieve uniform fibroblast colonization throughout the scaffold (Vieira et al., 2024), while simultaneously enhancing angiogenesis and extracellular matrix deposition (Guan et al., 2025).

Furthermore, optimizing pore size can alleviate inflammatory responses and extend the functional lifespan of implants (Li et al., 2015). For example, the bredigite scaffold, characterized by an ordered 3D-printed structure and uniform pore size distribution, promotes M2 macrophage polarization, thereby fostering an immune microenvironment conducive to bone regeneration (Xuan et al., 2023).

Porosity connectivity significantly influences the efficiency of material transport; scaffolds with high connectivity, such as microporous cross-linked particle structures, can establish a continuous network for nutrient and cell transport. A notable example is the microporous scaffold loaded with heparin micro-islands, which markedly enhanced cell infiltration and angiogenesis after 1 month of implantation (Nicklow et al., 2025).

However, it is essential to note that optimizing material morphology requires a careful balance with mechanical property requirements. While increasing porosity and pore size benefits cell infiltration and macrophage polarization phenotypes, it inevitably compromises the mechanical strength of the scaffold. Therefore, the advantages and disadvantages must be thoroughly evaluated in the design process.

### 4.2 Modulation of immunity by material degradation components

#### 4.2.1 Bioactive ions

Bioactive ions released from bone substitute materials, such as Ca^2+^, Mg^2+^, Si^4+^, Zn^2+^, and Cu^2+^, play a crucial role in modulating immune cell functions. These ions reshape the local immune microenvironment by influencing the phenotype, migration, differentiation, and cytokine release of immune cells, thereby synergistically promoting bone regeneration and enhancing the biocompatibility of implants.

Ca^2+^, as a major component of the bone matrix, play a critical role not only in the osteogenesis process but also in the modulation of immune responses via the calcium-sensing receptor (CaSR). An optimal concentration of Ca^2+^ activates the Wnt/β-catenin pathway and works synergistically with the PI3K/AKT and cAMP-PKA signaling pathways to promote the polarization of macrophages towards the M2 anti-inflammatory phenotype, thus inhibiting the release of pro-inflammatory factors such as TNF-α and IL-1β (Zhang et al., 2021; Shi et al., 2024). Furthermore, Ca^2+^ mimics the electrical properties of bone collagen fibers within a nanoscale heterogeneous electrical microenvironment, which modulates calcium ion channels, enhances the anti-inflammatory polarization of macrophages, and stimulates the secretion of factors such as FGF2. This process couples angiogenesis with immune regulation, thereby creating favorable conditions for bone regeneration (Tang et al., 2025).

Mg^2+^ plays a unique role in osteoimmunomodulation by synergistically modulating the immune microenvironment through multiple pathways to promote osseointegration. On one hand, Mg^2+^ antagonizes the activation of the NLRP3 inflammasome, inhibiting caspase-1 activation and the maturation of IL-1β, thereby alleviating local inflammatory responses (Wang et al., 2024; Ginsberg et al., 2017). The specific mechanism by which Mg^2+^ antagonizes NLRP3 inflammasome activation may involve the regulation of intracellular potassium efflux or the inhibition of mitochondrial reactive oxygen species; however, further research is needed (Wang et al., 2024). On the other hand, Mg^2+^ promotes the polarization of macrophages towards the M2 phenotype, enhancing angiogenesis and the release of anti-inflammatory factors through the PI3K/AKT signaling pathway, thereby improving the bone healing environment (Cheng et al., 2022; Wang et al., 2022). Moreover, Mg^2+^ demonstrates particularly pronounced effects under hyperglycemic pathological conditions. It alleviates oxidative stress by activating SESN2 expression in endothelial cells and promoting nuclear factor erythroid 2-related factor 2 (Nrf2) nuclear translocation, thus enhancing endothelial cell function and accelerating vascularization and bone repair in diabetic states (Liu L. et al., 2024). Collectively, these mechanisms endow Mg^2+^ with significant immunomodulatory and osteogenic potential.

Si^4+^ collaboratively modulates the immune microenvironment and the process of bone regeneration through multiple pathways. It not only promotes the secretion and adsorption of ECM components, such as Fn, but also facilitates the binding of Fn to integrin αv (Hung et al., 2014). This interaction induces conformational changes in the transmembrane receptor, subsequently activating focal adhesion kinase (FAK) and initiating downstream signaling cascades, including MAPK and PI3K/Akt. These cascades drive macrophage polarization towards the M2 phenotype and promote osteogenic differentiation. (Hung et al., 2014; Chen Y. et al., 2024). Additionally, Si^4+^ significantly inhibits NF-κB activation and the expression of downstream pro-inflammatory factors, such as TNF-α, IL-1β, and IL-6, effectively alleviating inflammatory responses (Hosseinpour et al., 2021; Peanlikhit et al., 2022). Furthermore, Si^4+^ enhances angiogenesis by promoting VEGF secretion and the formation of endothelial cell tubular structures (He et al., 2018). It synergizes with active components, such as Mg^2+^, to optimize vascularization and osteogenesis processes (Liu G. et al., 2024). In material applications, silicon-doped biomaterials, including bioactive glass and BCP scaffolds, demonstrate excellent performance in inhibiting inflammation, promoting osteogenesis, and enhancing angiogenesis through the controlled release of Si^4+^ (Cheng et al., 2022; Lu et al., 2024).

Zn^2+^, a prevalent bioactive ion, not only exhibits antibacterial properties but also promotes bone regeneration by coordinating immune homeostasis through various pathways. Firstly, as a crucial component of antioxidant enzymes such as superoxide dismutase (SOD) (Lokesha et al., 2025), Zn^2+^ effectively scavenges ROS and maintains intracellular zinc homeostasis by regulating metallothionein (MT) expression (Yang et al., 2022), thereby protecting immune cells from oxidative damage. Secondly, Zn^2+^ significantly inhibits the secretion of pro-inflammatory factors, including TNF-α and IL-6, which reduces inflammatory marker levels and alleviates pathological inflammatory responses (Lokesha et al., 2025; Giacconi et al., 2017). At the level of immune cell modulation, Zn^2+^ enhances the expression of osteogenesis-related genes by activating the transcription factor NF-κB, facilitates T cell proliferation and differentiation, improves the antigen-presenting capacity of DCs (Deng et al., 2024), and optimizes the synergistic efficacy of innate and adaptive immune responses (Schuhladen et al., 2020). It is noteworthy that the surface modification of Zn-Li alloys through calcium plasma immersion ion implantation (PIII) technology significantly promotes the release of Zn^2+^ ions and accelerates material degradation, thereby enhancing its osteogenic and angiogenic properties (Li X. et al., 2025). This systematic modulation, ranging from molecular antioxidant protection to cellular function activation, provides critical immunoregulatory targets for the design of bone substitute materials.

Cu^2+^ plays a crucial immunomodulatory role in bone substitute materials, particularly regarding its antibacterial, anti-inflammatory, and pro-angiogenic properties. Generally, low concentrations of Cu^2+^ (100 μM) induce macrophage polarization towards the M1 phenotype, exacerbating inflammatory responses (Díez-Tercero et al., 2021; Huang et al., 2019). Notably, while this M1 polarization induced by high concentrations of Cu^2+^ and its accompanying pro-inflammatory state is generally detrimental to tissue repair, it can be actively utilized to enhance the antibacterial efficacy of the material by combating early-stage infections and eliminating pathogens. For instance, the Cu-doped micro/nano-topological structured surface (Cu-Hier-Ti) induces M1 polarization by activating copper transport signals (CTR1 and ATP7A), synergistically enhancing the material’s antibacterial and anti-inflammatory efficacy (Huang et al., 2019). Regarding angiogenesis, Cu^2+^ significantly upregulates the expression of angiogenic factors such as VEGF and bFGF, promoting endothelial cell proliferation and migration, and accelerating the construction of functional vascular networks (Li et al., 2023). Additionally, the time-sequenced release of Cu^2+^ enables dynamic immune modulation. In the Cu-Sr bilayer bioactive glass nanoparticles (CS-BGNs) system, the early rapid release of Cu^2+^ effectively controls inflammation and infection, while the later sustained release of Sr^2+^ shifts towards promoting osteointegration. This spatiotemporally precise ion delivery provides an innovative solution for optimizing bone repair (Wu et al., 2023).

Bioactive ions such as Ca^2+^, Mg^2+^, Si^4+^, Zn^2+^, and Cu^2+^ have demonstrated significant potential in modulating immune cell functions and optimizing the microenvironment for bone regeneration. However, their clinical translation faces critical challenges. The primary challenge is the insufficient in-depth analysis of molecular mechanisms, particularly the unclear signal transduction pathways through which Mg^2+^ antagonizes the NLRP3 inflammasome (Wang et al., 2024), Additionally, the release of ions lacks precise spatiotemporal control, and fluctuations in local concentration—such as the pro-inflammatory risks induced by high concentrations of Cu^2+^—can lead to uncontrollable immunomodulatory effects (Huang et al., 2019). Furthermore, there is a notable gap in research regarding the mechanisms of synergistic effects among multiple ions, exemplified by the lack of evidence for the synergistic immunomodulatory effects of combinations like Zn^2+^/Sr^2+^ (Zhong et al., 2022). To address these bottlenecks, future research should prioritize: the in-depth elucidation of the intricate molecular mechanisms through which ions regulate immune cell polarization via key signaling pathways such as NF- κB and PI3K-AKT-mTOR (Liang et al., 2024; Sun H. et al., 2021); the development of intelligent responsive delivery systems to achieve dynamic optimization of local ion concentrations and on-demand release (Li et al., 2023); and the systematic exploration of synergistic regulatory strategies involving multiple ions such as Mg^2+^/Cu^2+^ (Zhong et al., 2022; Lourenco et al., 2019). Breakthroughs in these areas will drive the design of a new generation of bone substitute materials, ultimately enhancing bone repair efficacy and long-term stability through the synergistic optimization of the immune microenvironment.

#### 4.2.2 Material degradation products

**FIGURE 4:**
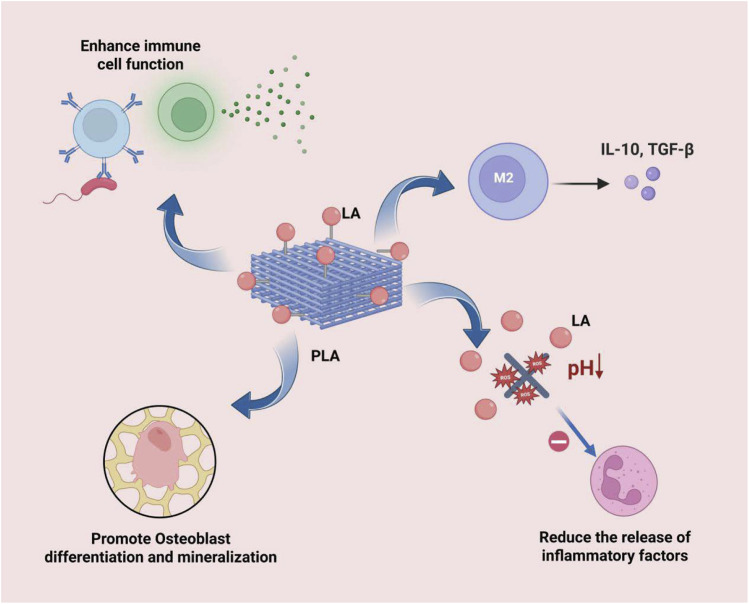
LA can effectively induce the polarization of macrophages towards the M2 type, inhibit the activity of inflammatory cells and the release of their pro-inflammatory mediators, directly stimulate the differentiation and mineralization of osteoblasts, and regulate the functions of immune cells, thereby enhancing the body’s systemic resistance to infections.

The degradation products of bone substitute materials, such as lactic acid (LA) released by PLA, play a crucial role in modulating immune responses and promoting tissue regeneration (Figure 4). These degradation products not only directly influence the microenvironment at the implantation site but also systemically regulate immune cell functions, thereby affecting the outcomes of tissue repair and regeneration. Studies have demonstrated that they modulate immunity through various mechanisms:

Firstly, LA can inhibit the antigen-presenting capacity of DCs and attenuate T cell activation through the GPR81 receptor, thereby modulating immune tolerance and reducing the risk of immune rejection after material implantation (Llibre et al., 2025). Additionally, it can significantly influence the phenotypic polarization of immune cells. For instance, metabolites such as LA can effectively induce the polarization of macrophages towards the M2 phenotype, which exhibits anti-inflammatory and pro-repair functions. This process is accompanied by the upregulation of anti-inflammatory cytokines, such as IL-10, which suppresses excessive inflammatory responses and promotes tissue healing (Ryma et al., 2021; Negi et al., 2024). It is particularly noteworthy that LA can also promote the expression of anti-inflammatory factors such as IL-10 and enhance M2 macrophage polarization through histone lactylation modification (Choi et al., 2025).

Secondly, the modulation of inflammatory responses by degradation products is of paramount importance. LA effectively inhibits the inflammatory response through the following mechanisms: Firstly, it enhances the activity of SOD, which accelerates the clearance of superoxide anions. Secondly, it improves the mitochondrial membrane potential and promotes ATP production, thereby reducing the leakage of mitochondrial ROS and inhibiting ROS production (Gu et al., 2024; Bustamante-Barrientos et al., 2025). These effects collectively suppress the activity of inflammatory cells and the release of pro-inflammatory mediators, ultimately leading to a significant alleviation of the inflammatory response (Chor et al., 2022; Liu H. et al., 2023). At the metabolic level, LA reprograms immune cells by influencing glycolysis and mitochondrial function. It inhibits CD8^+^ T cell function while promoting the proliferation of Tregs, thereby further enhancing the processes of immunosuppression and tissue repair (Choi et al., 2025; Iozzo et al., 2025).

Moreover, these immunomodulatory effects are directly linked to and promote the bone regeneration process. Metabolites such as LA not only create a favorable regenerative microenvironment through the aforementioned immunomodulation but also directly stimulate osteoblast differentiation and mineralization, inhibit osteoclast activity (Hong et al., 2023), and release angiogenic factors to facilitate the growth of new blood vessels, providing essential nutritional support for bone regeneration (Kong et al., 2024).

It is important to note that the immunomodulatory effects of degradation products extend beyond localized areas. Certain metabolites can enter the bloodstream and influence systemic immune status by modulating the functions of immune cells, such as lymphocytes (Yang B. et al., 2025), potentially enhancing the body’s overall anti-infection capabilities (Li et al., 2016).

Finally, the biocompatibility and long-term safety of degradation products are fundamental prerequisites for the successful application of materials. An ideal bone substitute material should promote cell proliferation and differentiation while inducing only minimal and controllable immune responses, thereby ensuring the long-term safety of the products (Tseng and Fang, 2023; Káčerová et al., 2023).

In summary, the degradation products of bone substitute materials, particularly LA, modulate the transition of macrophages to the M2 phenotype, inhibit local inflammation, promote the balance between osteoblasts and osteoclasts, stimulate angiogenesis, and influence systemic immune status through multiple mechanisms. These include the GPR81 signaling pathway, histone lactylation, and cellular metabolic reprogramming. Collectively, these mechanisms form the core immunomodulatory framework that facilitates bone tissue repair and regeneration. A deeper understanding of these mechanisms not only provides critical theoretical foundations for optimizing the design of bone substitute materials but also establishes an important basis for their clinical safety and efficacy. Future research should further elucidate the precise molecular pathways of interactions between degradation products and the immune system to guide the development of a new generation of bone substitute materials with enhanced immunomodulatory properties.

### 4.3 Biofunctionalization of material surfaces

The strategy of biofunctional modification on the surface of bone substitute materials has been demonstrated as an effective method to optimize the interaction between the material and host interface, precisely regulate local and systemic immune responses, and thus promote bone regeneration and integration. By introducing specific bioactive molecules or constructing functional topological structures on the material surface, the immune compatibility and osteoinductive properties of the materials can be significantly enhanced. It is noteworthy that these strategies possess considerable clinical significance in addressing the challenges associated with bone defect repair in patients exhibiting high-risk factors, such as metabolic diseases (e.g., diabetes) or infections. Specifically, the immobilization of proteins (such as bovine serum albumin) or growth factors on the material surface in a non-covalent manner, for instance, utilizing gelatin wet granulation technology to modify the surface of poly (methyl methacrylate) (PMMA), facilitates the sustained release of these biomolecules. This release effectively modulates the local immune microenvironment, suppresses excessive inflammation, and promotes osteoblast adhesion and the bone healing process (Oliveira et al., 2008). This strategy is particularly effective for repairing bone defects in diabetic patients. The diabetic microenvironment is frequently characterized by chronic inflammation and impaired angiogenesis. Controlled release of growth factors, such as VEGF and BMP-2, can effectively mitigate these pathological conditions. This approach synergizes with immunomodulatory effects to overcome the barriers to bone healing in diabetic conditions (Zheng et al., 2024). Similarly, the incorporation of bioactive substances (such as β-TCP) into hydrogel coatings (e.g., methacrylated gelatin, GelMA) or their immobilization onto substrates (such as 3D-printed polyether ether ketone, PEEK) through sulfonation treatment not only significantly enhances the adhesion, proliferation, and osteogenic differentiation of BMSCs but also actively modulates the phenotype and function of immune cells, such as macrophages, thereby establishing an immune homeostasis conducive to bone regeneration (Lin et al., 2025). This type of composite coating technology provides the potential for developing biomimetic implants with heterogeneous structures that more accurately mimic the chemical and physical properties of natural bone tissue. This advancement facilitates host tissue integration and mitigates the risk of implantation failure (Chen J. et al., 2024). Secondly, nanoscale surface structure engineering can significantly enhance the biological activity of materials. For instance, the construction of a nano-oxide layer on the surface of titanium particles through electrochemical anodization and heat treatment results in a nanostructured surface that can induce the formation of HA when immersed in simulated body fluid. The formed HA can regulate the recruitment and function of immune cells through its surface chemical properties and topological structure, thereby promoting osseointegration by optimizing the immune microenvironment (Özcolak et al., 2024; Karaji et al., 2016). This regulation is crucial for enhancing the long-term stability of load-bearing implants, such as joint replacements and dental implants (Lee et al., 2025). Additionally, the integration of glycosaminoglycans (such as heparin) into the material matrix (such as mineralized collagen) allows for precise control over protein adsorption behavior, influencing the fate of mesenchymal stem cells (MSCs) and the local immune microenvironment (König et al., 2014). Photofunctionalization technology effectively improves the hydrophilicity and biological activity of materials, promoting the response of bone regeneration-related cells. For example, ultraviolet treatment can create a titanium dioxide coating on the surface of HA, achieving biological functionalization of the material surface. When combined with advanced technologies such as tannic acid antibacterial coating and RNA interference, this approach can also impart significant anti-infective capabilities to the material, clearing pathogens while mitigating the excessive immune response triggered by infection, thus creating a clean microenvironment for osseointegration (Liu Z. et al., 2024; Kim et al., 2019). This surface treatment technology, which integrates both antibacterial and immunomodulatory functions, presents a novel solution for addressing challenging clinical issues, such as infectious nonunion and osteomyelitis (Zhang H. et al., 2025). Furthermore, fluorinated surface modification has been demonstrated to significantly enhance the osseointegration performance of implants, such as by increasing the bone-implant contact rate and stability, with a mechanism partly attributed to its favorable modulation of local immune responses (Dasmah et al., 2014).

In summary, the surface biofunctionalization modifications of bone substitute materials—including biomolecular coating, nanostructure design, glycosaminoglycan integration, photofunctionalization, and antibacterial/fluorination treatment—constitute the core strategy to enhance their efficacy in osteoimmunomodulation. These modifications collectively create an immune microenvironment that is conducive to bone tissue repair and regeneration by precisely intervening in immune cell behavior, modulating inflammatory processes, promoting osteoblast activity, and inhibiting infections. These strategies hold significant promise in addressing critical clinical bottlenecks, including the modulation of abnormal bone healing microenvironments in systemic diseases such as diabetes, the prevention and treatment of implant-related infections, and the enhancement of long-term stability of load-bearing implants in osteoporotic bone matrices. A comprehensive understanding and optimization of these surface engineering strategies are crucial for the development of a new generation of bone substitute materials with intelligent immune modulation capabilities. Future research should prioritize the evaluation of the long-term *in vivo* effects of these modification techniques, the dynamic changes in immune responses, and their translational potential in complex clinical scenarios.

### 4.4 Intelligent delivery of immunomodulatory factors

The osteoimmune microenvironment comprises a diverse array of signaling molecules and cytokines that directly influence the processes of osteogenesis and osteoclastogenesis by regulating the immune system. Consequently, bone substitute materials can effectively modulate local immune responses through the loading and delivery of immunomodulatory factors, thereby enhancing bone tissue regeneration and functional reconstruction.

The surface loading of cytokine coatings on materials represents a common and effective strategy for osteoimmunomodulation (Gong et al., 2020). Once the material is implanted in the body, the cytokines released from the coating directly or indirectly regulate the osteogenesis process by modulating macrophage polarization and the RANK/RANKL signaling pathway (Xie et al., 2020). For example, Kara’s team developed an osteoimmunomodulatory scaffold with sequential release properties of IFN-γ and IL-4 in a mouse subcutaneous implantation model (Wang M. et al., 2016). This scaffold achieved precise modulation of angiogenesis by rapidly releasing IFN-γ to induce macrophage polarization towards the M1 phenotype, followed by the sustained release of IL-4 to promote the transformation to the M2 phenotype. Notably, in addition to its role in macrophage polarization, IL-4 can synergize with IL-33 to inhibit osteoclast function (Amarasekara et al., 2018): both cytokines promote the differentiation of monocytes into DCs and macrophages, thereby downregulating the differentiation potential of osteoclast precursor cells and interfering with osteoclastogenesis (Zaiss et al., 2011). In addition to the aforementioned cytokines, IL-10, IL-12, and the interferon family also play significant roles in the modulation of bone metabolism. IL-10 effectively inhibits the bone resorption process by suppressing the expression of nuclear factor of activated T cells cytoplasmic 1 (NFATc1), a core regulatory element of osteoclast differentiation (Tanaka et al., 2019). Members of the IFN family, including IFN-α, IFN-β, and IFN-γ, exhibit inhibitory effects on osteoclast differentiation: IFN-α/β primarily participates in the modulation of innate immunity (Hu et al., 2023), while IFN-γ, in addition to activating macrophage functions, can also inhibit osteoclast differentiation through the negative modulation of the RANK/RANKL/OPG signaling pathway (Takayanagi et al., 2000). In summary, the modulation of the bone immune microenvironment through the combination of various cytokines and time-controlled release to promote osteogenesis has become an important research strategy for the development of novel bone substitute materials.

Pharmacological studies have demonstrated that various small-molecule drugs can modulate the osteogenic process through their osteoimmunomodulatory functions. With advancements in biomaterial processing technologies, these drugs can be endowed with osteoimmunomodulatory properties via surface modifications. A notable example is the zinc finger-inspired peptide-metal-phenolic nanocoating developed by researchers, including Xu et al. (Xu et al., 2024). This system utilizes zinc ion-phenolic coordination to achieve stable loading of the small-molecule drug Abaloparatide (ABL) and enables a sustained release for over 7 days. Experiments have confirmed that the ABL-loaded zinc-phenolic network (ABL@ZnTA) significantly enhances the polarization of macrophages towards the M2 phenotype compared to the unloaded control group. This shift induces an immune microenvironment conducive to bone regeneration and promotes the osteogenic differentiation of BMSCs. Furthermore, rapamycin has been shown to facilitate M2 polarization of macrophages by inducing autophagy, thereby alleviating inflammatory responses and enhancing osteogenesis (Yurube et al., 2024; Huang X-R. et al., 2025). Building on this, the rapamycin-loaded virus-like hollow silica nanoparticles (R@HSNs) developed by Zhang et al. were found to be phagocytosed by macrophages and transported to lysosomes, triggering autophagy-mediated M2 polarization, which significantly promoted bone regeneration in a mouse calvarial defect model (Zhang Q. et al., 2023). This system also optimized the osteoimmune microenvironment by synergistically downregulating pro-inflammatory factors (IL-6, IL-1β, TNF-α) and upregulating anti-inflammatory markers (CD163, CD206, IL-10).

Natural small molecule substances, such as chitosan and hyaluronic acid, have been extensively utilized in the surface functionalization of bone substitute materials due to their remarkable biocompatibility and immunomodulatory properties. Chitosan, a natural polysaccharide, has been shown to inhibit inflammation and promote repair by driving macrophage polarization towards the anti-inflammatory M2 phenotype (Guo et al., 2018). Concurrently, hyaluronic acid interacts with receptors such as CD44 on the surface of immune cells, inhibiting neutrophil migration and promoting M2 polarization, thereby regulating inflammation levels and facilitating tissue repair (Guo et al., 2024; Luo et al., 2024; Salathia et al., 2023). Notably, the combination of chitosan and hyaluronic acid can synergistically inhibit the expression of pro-inflammatory factors TNF-α and IL-6 while promoting the secretion of the anti-inflammatory factor IL-10, thus remodeling the inflammatory microenvironment. Furthermore, hyaluronic acid modification can significantly reduce the immunogenicity of chitosan nanoparticles by decreasing serum protein adsorption, thereby mitigating excessive immune responses (Almalik et al., 2017; Ciołek et al., 2024). In bone repair applications, this composite system not only enhances the osteogenic differentiation capacity of BMSCs, as evidenced by increased ALP activity and calcium deposition, but also significantly promotes bone defect repair in animal models, particularly when combined with MSCs, demonstrating a synergistic effect on bone regeneration (Abazari et al., 2019).

In summary, current research on the regulatory mechanisms of bone metabolism emphasizes the targeted delivery of osteoimmunomodulatory factors, such as cytokines, small molecule drugs, and natural polysaccharides, through material design. This approach aims to coordinate macrophage polarization states and regulate the RANK/RANKL/OPG signaling pathway. While this strategy significantly enhances the construction of the osteoimmune microenvironment, critical knowledge gaps persist regarding the underlying molecular mechanisms, highlighting the need for further research.

### 4.5 Bionic and intelligent responsive materials

Bionic and intelligent responsive bone substitute materials provide a more efficient and precise solution for bone repair by mimicking the structure and function of natural bone while integrating intelligent response mechanisms.

The design of bionic bone substitute materials fundamentally revolves around the accurate simulation of the microstructure and biomechanical properties of natural bone (Hsu et al., 2025). To achieve this, 3D printing technology can be employed to control the porosity and topological configuration of scaffolds, thereby facilitating structural bionics. Specifically, regarding pore structure design, triangular pore structures exhibit superior mechanical stability, outperforming rectangular, honeycomb, and diamond configurations (Lv et al., 2023). In terms of material selection, HA and polycaprolactone (PCL) composites demonstrate excellent biocompatibility and mechanical properties due to their bone-like chemical composition (Rezaei and Mohammadi, 2013). At the functional bionic level, the integration of tea polyphenol-magnesium (TP-Mg) nanoparticles into α/β-tricalcium phosphate (α/β-TCP) scaffolds can simultaneously achieve antibacterial, anti-inflammatory, and osteoinductive effects, significantly promoting the repair of infectious bone defects (Hu X. et al., 2024). A more in-depth functional simulation is exemplified by the periosteum biomimetic strategy, which involves using pre-osteoblast-derived matrix (pODM) to coat the hydrogel system, effectively reconstructing the bone formation microenvironment and enhancing bone regeneration efficacy (Yu et al., 2020). Currently, biomimetic bone substitute materials exhibit great potential in clinical applications; for instance, 3D printing technology can rapidly fabricate biomimetic bone scaffolds with complex internal structures, thereby meeting the needs of personalized treatment (Bisht et al., 2021).

Intelligent responsive bone substitute materials represent a class of advanced materials capable of automatically adjusting their functions in response to specific environmental stimuli, such as pH, enzyme activity, and ROS. These materials exhibit significant potential in the fields of bone tissue engineering and drug delivery, particularly in the treatment of bone infections, tumors, and inflammation-related diseases (Chaudhari et al., 2024). pH-responsive bone substitute materials achieve precise immune modulation through intelligent release mechanisms and microenvironment-responsive characteristics. The core principle involves modulating drug release kinetics based on local pH variations; for instance, HA nanocrystals remain stable at physiological pH but accelerate dissolution and release therapeutic agents in acidic microenvironments, such as inflammatory or tumor sites, thereby achieving targeted effects and reducing systemic toxicity (Lelli et al., 2016). Similarly, vancomycin-loaded ZIF8 nanocrystals (ZIF8/VAN) demonstrate a significantly higher release rate at pH 5.4 compared to pH 7.4 conditions. This characteristic facilitates precise targeting of the acidic environment at infection sites, effectively inhibiting the proliferation of *Staphylococcus aureus* (Karakeçili et al., 2019). At the level of immune modulation, pH-responsive materials can further optimize the microenvironment by scavenging ROS and inhibiting the generation of inflammatory mediators. For instance, biomimetic nanosystems can release anti-inflammatory agents on demand through pH sensing, significantly alleviating the inflammatory response of human periodontal ligament stem cells and promoting tissue repair (Chen et al., 2022). Moreover, surface engineering significantly enhances the functional diversity of materials. The surface of the Ti6Al4V alloy, when subjected to chemical-thermal treatment, demonstrates pH-dependent wettability and adhesion properties. This characteristic enables efficient loading of synthetic peptides, facilitating controlled drug release and antibacterial functions (Rodriguez et al., 2020). This multifunctional feature, which integrates targeted delivery, immune modulation, and interface optimization, offers an innovative solution for the treatment of bone infections and the repair of defects.

The core design concept of enzyme activity-responsive bone substitute materials revolves around their capacity to specifically detect changes in the activity of crucial enzymes, such as MMPs, cathepsins, and other enzymes associated with inflammation and remodeling, within the bone repair microenvironment (Zong et al., 2024). This sensing capability allows for the dynamic modulation of their behavior. For example, through the cleavage of enzyme-sensitive bonds, these materials can facilitate on-demand degradation or the precise release of encapsulated immunomodulatory factors (Zong et al., 2024; Dong et al., 2024). This enzyme-responsive intelligent release mechanism empowers the material to react to increased specific enzyme activity during the inflammatory phase, thereby delivering immunomodulatory signals in a timely and localized manner. Consequently, it actively intervenes in macrophage phenotype polarization, mitigates excessive inflammatory responses, and fosters the development of a reparative microenvironment. Ultimately, it achieves a spatiotemporally specific immunomodulatory function that aligns more closely with the dynamic requirements of bone regeneration, significantly differentiating it from the static drug release or degradation mechanisms of traditional materials.

Extensive bone defects, particularly those resulting from trauma or infection, necessitate the use of bone substitute materials due to the limited natural healing capacity of bone. However, persistent inflammatory responses and elevated levels of ROS at the defect site significantly impede the bone regeneration process (He et al., 2024). To address this challenge, researchers have developed an injectable dynamic hydrogel matrix (HAC). This hydrogel is composed of hyaluronic acid-functionalized dopamine, a 4-formylphenylboronic acid crosslinker, and carboxymethyl chitosan. The innovation of this system lies in its incorporation of dimethyl fumarate (DMF), which exhibits anti-inflammatory and antioxidant properties, thereby forming a ROS-responsive hydrogel (DHAC). The borate ester bonds in DHAC can specifically cleave in response to high ROS microenvironments, enabling the precise and on-demand release of DMF (Huang Q. et al., 2025). Physicochemical characterization reveals that DHAC possesses excellent injectability and self-healing capabilities, allowing it to form stable three-dimensional scaffolds at defect sites while precisely releasing DMF in response to local ROS levels. In terms of immune modulation, DHAC effectively reverses the polarization of M1 macrophages by scavenging intracellular ROS and inhibiting the secretion of pro-inflammatory factors such as TNF-α and IL-6. This significantly alleviates the inflammatory cascade and creates a favorable immune microenvironment for bone regeneration. Through an integrated “sensing-release-modulation” design, this ROS-responsive hydrogel system simultaneously addresses three core challenges in bone regeneration: inflammation control, oxidative stress elimination, and osteogenesis promotion. It offers a novel strategy that combines intelligence and functionality for the treatment of complex bone defects. Notably, in complex clinical cases, like the post-surgical defect after resection of osteosarcoma, a material able to inhibit the recurrence of the tumour and stimulate bone regeneration is needed. Zn-based porous scaffolds made by additive manufacturing which are biodegradable, like Zn-0.8Li IPC scaffolds with a Gyroid unit, are an important improvement. The scaffolds released Zn^2+^ and Li^+^ ions in a suitable ratio during degradation while showing a noteworthy anti-tumor effect. Namely, they impaired the proliferation and migration of osteosarcoma cells while enhancing apoptosis. They also enhanced osteogenic differentiation *in vitro* and promoted bone regeneration *in vivo*. Transcriptomic analyses suggest the dual functionality occurs via downregulation of the PI3K/Akt signaling pathway, indicating the intelligent responsiveness of the scaffold towards the tumor microenvironment and its substantial translational potential against postoperative osteosarcoma along with the repair of related bone defect (Lu et al., 2025).

In summary, the research in this field is continuously advancing bone tissue engineering, from biomimetic design to intelligent response mechanisms and clinical applications. In the future, with technological advancements, these materials are expected to play a significant role in more complex scenarios of bone defect repair, providing better treatment outcomes for patients (Yu et al., 2025).

## 5 Conclusions and future perspectives

The development of bone substitute materials grounded in the concept of osteoimmunomodulation continues to encounter multifaceted challenges in both fundamental science and clinical translation. At the level of immunomodulatory mechanisms, the host’s response to implanted materials is characterized by a high degree of complexity, arising from the cascade reactions of the innate and adaptive immune systems, the plasticity of immune cell subsets, and significant variations in genetic backgrounds and immune states among individuals. Regarding material-host interactions, the dynamically changing local microenvironmental parameters—including, but not limited to, pH gradients, oxidative stress levels, mechanical stimuli, and fluctuations in cytokine concentrations—collectively influence the degradation kinetics and immunomodulatory efficacy of the materials. The long-term safety evaluation system remains incomplete, particularly due to a lack of systematic research on the long-term biocompatibility of novel immunomodulators such as exosomes, cell membrane coatings, and genetically modified materials. Numerous obstacles hinder the clinical translation pathway, including the development of large-scale production processes compliant with GMP standards, the long-term maintenance of material stability, the establishment of stringent quality control systems, and the validation of clinical efficacy through multicenter clinical trials. These challenges represent critical translational barriers from basic research to clinical application.

To address the aforementioned challenges and advance the transformation process, we recommend prioritizing the following categories of smart osteoimmunomodulation materials, which already have strong preclinical evidence, for multi-center clinical trials: (1) multifunctional bioceramic composites with sequential ion release capabilities, such as combinations of Mg^2+^/Si^4+^ and Cu^2+^/Sr^2+^; (2) hydrogel systems loaded with specific immunomodulatory factors, including IL-4, IL-10, and TGF-β, that exhibit inflammation-responsive release characteristics; (3) 3D-printed polymer composite scaffolds that are specifically biofunctionalized on the surface, incorporating biomimetic coatings and specific topological structures. Concurrently, there is an urgent need to establish a standardized evaluation system for bone immune materials, which should encompass: (i) standardized characterization processes for the physicochemical properties and degradation behaviors of materials; (ii) standardized evaluation models for *in vitro* immunomodulatory efficacy, such as quantitative analysis of macrophage polarization profiles and lymphocyte subgroup co-culture systems; (iii) multimodal evaluation standards for the bone immune microenvironment and regenerative effects in large animal bone defect models, including histology, micro-CT, immunohistochemistry, and cytokine profiling; (iv) a tracking assessment plan for systemic immune responses and biosafety after long-term implantation.

The development of future bone substitute materials is expected to exhibit an innovative trend characterized by interdisciplinary integration. The concept of precision medicine will guide the design of individualized materials, allowing for precise analysis of patient-specific immune microenvironments through the integration of single-cell multi-omics analysis, spatial transcriptomics, and AI-assisted modeling. This approach will facilitate the creation of customized immune modulation strategies. Intelligent responsive materials are anticipated to emerge as a research hotspot, particularly in the development of four-dimensional printed scaffolds that can sense and adapt to changes in the local microenvironment. These materials are capable of dynamically regulating immune cell polarization and stem cell differentiation in accordance with the biological requirements of various stages of bone repair. Cell engineering technology is poised for significant breakthroughs, including the optimization of macrophage polarization protocols *in vitro*, the development of CRISPR-based gene editing techniques for immune cells, and the establishment of a controllable cytokine sustained-release system. In terms of manufacturing technology, microfluidic-assisted bioprinting will enable the precise construction of vascularized bone tissue, while organ-on-a-chip technology will provide a high-throughput platform for material screening. The synergistic development of these innovative directions will advance bone substitute materials from passive structural replacements to active immune modulation and functional regeneration, ultimately achieving true bone tissue engineering reconstruction.

In summary, research on bone substitute materials that leverage osteoimmunomodulatory properties is experiencing a paradigm shift from passive “immune silence” to active “immune dialogue.” This review systematically elucidates the fundamental principles of osteoimmunology, highlights the core regulatory role of the immune system in bone repair, and comprehensively summarizes the latest advancements in achieving precise modulation of the immune microenvironment through strategies such as modulation of material physicochemical properties, surface functionalization, and the delivery of bioactive factors. Despite challenges related to the complexity of immune responses, long-term material safety, and clinical translation, the development of cutting-edge technologies such as single-cell techniques, smart responsive materials, and cell engineering is poised to enable the next-generation of bone substitute materials with immunomodulatory functions to transition from mere structural replacement to functional regeneration. Future research should prioritize the establishment of standardized evaluation systems, foster deep interdisciplinary integration, accelerate the development of innovative materials with clinical translation potential, and offer more effective treatment strategies for bone defect repair. Breakthroughs in this field will not only propel advancements in bone regenerative medicine but also provide valuable insights for the development of other tissue engineering materials.
